# The efficacy of Advantix^®^ to prevent transmission of *Ehrlichia canis* to dogs by *Rhipicephalus sanguineus* ticks

**DOI:** 10.1051/parasite/2013037

**Published:** 2013-10-21

**Authors:** Josephus Johannes Fourie, Herman Gerhardus Luus, Dorothee Stanneck, Frans Jongejan

**Affiliations:** 1 ClinVet International (Pty) Ltd PO Box 11186 Universitas, Bloemfontein 9321 South Africa; 2 Bayer Animal Health GmbH, BHC Business Group Animal Health, Clinical Development, Building 6700 51368 Leverkussen Germany; 3 Utrecht Centre for Tick-Borne Diseases (UCTD), Faculty of Veterinary Medicine, Utrecht University, Yalelaan 1 3584 CL Utrecht The Netherlands; 4 Department of Veterinary Tropical Diseases, Faculty of Veterinary Science, University of Pretoria, Private Bag X04, Onderstepoort 0110 South Africa

**Keywords:** Imidacloprid, Permethrin, *Ehrlichia canis*, *Rhipicephalus sanguineus*, Ehrlichiosis, Transmission

## Abstract

The capacity of a topical combination of imidacloprid and permethrin (Advantix^®^) to prevent transmission of *Ehrlichia canis* was studied in two groups of six dogs. One group served as controls, whereas the other group was treated. All dogs were exposed to *E. canis*-infected *Rhipicephalus sanguineus* ticks on Days 7, 14, 21 and Day 28 post acaricidal treatment. The adult *R. sanguineus* ticks were released into the individual kennels of the dogs to simulate natural tick exposure. *In situ* tick counts were conducted on Day 9, 16 and 23 and any remaining ticks were counted and removed on Day 30. The efficacy of the acaricidal treatment against *R. sanguineus* ranged between 96.1% and 98.9% at 48 h post-application and lasted up to 4 weeks. Four out of six control dogs became infected with *E. canis*, as demonstrated by the presence of specific *E. canis* antibodies and the detection by PCR of *E. canis* DNA in blood samples. These dogs became thrombocytopenic and displayed fever and were consecutively rescue-treated by doxycycline. None of the six treated dogs became infected with *E. canis,* as confirmed by the lack of specific antibodies and absence of *E. canis* DNA in blood samples. Advantix^®^ prevented transmission of *E. canis* and provided protection against monocytic ehrlichiosis for 4 weeks post acaricidal treatment.

## Abstract (fr)

La capacité d’une association locale d’imidaclopride et perméthrine (Advantix^®^) pour prévenir la transmission d’Ehrlichia *canis* a été étudiée dans deux groupes de six chiens. Un groupe a servi de témoin, tandis que l’autre groupe a été traité. Tous les chiens ont été exposés à des tiques *Rhipicephalus sanguineus* infectées par *E. canis* aux jours 7, 14, 21 et 28 jours après traitement acaricide. Les *R. sanguineus* adultes ont été lâchés dans les niches individuelles des chiens pour simuler une exposition naturelles aux tiques. Des comptages de tiques *in situ* ont été menés aux jours 9, 16 et 23 et les tiques restantes ont été comptées et enlevées au jour 30. L’efficacité du traitement acaricide contre *R. sanguineus* a varié entre 96,1 % et 98,9 % à 48 h après l’application et a duré jusqu’à quatre semaines. Quatre des six chiens témoins ont été infectés avec *E. canis*, comme en témoigne la présence d’anticorps spécifiques contre *E. canis* et la détection par PCR d’ADN d’*E. canis* dans les échantillons de sang. Ces chiens sont devenus thrombocytopénique et fiévreux et ont été consécutivement traités jusqu’à guérison par la doxycycline. Aucun des six chiens traités n’a été infecté par *E. canis*, comme le confirme l’absence d’anticorps spécifiques et d’ADN d’*E. canis* dans les échantillons de sang. Advantix^®^ a empêché la transmission d’*E. canis* et a fourni une protection contre l’ehrlichiose monocytaire pendant quatre semaines après le traitement acaricide.

## Introduction

Ticks and tick-borne diseases that affect the health of dogs are expanding in different regions of the world [5]. Changes in human behaviour (increased outdoor recreation and international travel with companion animals) and changes in landscape ecology with increased wildlife host abundance for ticks are among the factors contributing to the increased incidence of canine tick-borne diseases [2]. Worldwide, canine monocytic ehrlichiosis, babesiosis and granulocytic anaplasmosis are the most common tick-borne diseases of dogs [13, 17].

The capacity of ticks to transmit these protozoan or bacterial pathogens varies widely. For instance, the cosmopolitan tick, *Rhipicephalus sanguineus*, is vector of *Ehrlichia canis,* which is the cause of canine monocytic ehrlichiosis. Moreover, *R. sanguineus* transmits a broad range of other pathogens, including *Babesia vogeli*, *Babesia gibsoni*, *Hepatozoon canis*, *Rickettsia conorii*, *Rickettsia rickettsii* and probably also *Anaplasma platys*, the cause of thrombocytic anaplasmosis [5].

Effective acaricidal control of ticks is a necessity in many parts of the world. Acaricidal treatment that kills ticks reduces the number of ticks capable of transmitting tick-borne pathogens.

In addition, the effectiveness of an acaricide that acts sufficiently fast to prevent transmission of tick-borne pathogens carries an important added value that needs to be demonstrated empirically. Studies that have been conducted thus far suggest that topically applied acaricides can assist in the prevention of the transmission of specific tick-borne pathogens. For instance, the ability of imidacloprid/permethrin to prevent transmission of *Anaplasma phagocytophilum*, the cause of granulocytic anaplasmosis, from naturally infected *Ixodes scapularis* ticks to dogs was reported several years ago [3].

A relatively new area of research is the development of transmission-blocking models, wherein the ability of tick control compounds to prevent transmission of pathogens can be effectively measured under controlled laboratory conditions [14]. First models were established for the blocking of transmission of *Babesia canis* by infected *Dermacentor reticulatus* ticks [9, 14]. Recently, further developments in this area have been the creation of a tick exposure laboratory model, wherein transmission of *E. canis* by infected *R*. *sanguineus* ticks can be studied [10]. Since the mode of action of compounds differs, it is necessary to evaluate each product’s ability to prevent transmission of pathogens. For example, some products may have an irritant and toxic effect, such as permethrin and other substances such as amitraz present an effect which includes both an expellant effect, along with acaricidal properties [4, 8].

Here, this model was used to evaluate the efficacy of the topical product Advantix^®^, which combines imidacloprid 10% w/v with permethrin 50% w/v, in preventing the transmission of *E. canis* by infected *R. sanguineus* ticks to dogs.

## Material and methods

### Study design

This study was in compliance with the animal welfare requirements and carried out according to International Cooperation on Harmonization of Technical Requirements for Registration of Veterinary Medicinal Products Guideline 9: Good Clinical Practice [6]. It was a randomised, blinded efficacy study conducted with two groups of six dogs, which were males and females of mixed breed (mongrel), with a weight range of 13.8–18.8 kg. All dogs were kept individually in tick-proof kennels and were observed twice daily for health abnormalities. The dogs, all negative for *E. canis* specific antibodies in the indirect fluorescent antibody test (IFA), were ranked according to weight and divided randomly into two equal groups. Group 1 dogs were designated control and Group 2 dogs were treated with 10% w/v imidacloprid and 50% w/v permethrin on Day 0. The product was administered according to label instructions (topical spot-on at four spots along the back from the shoulder to the base of the tail) to each of the dogs in the acaricidal treatment group.

### Infection of *R. sanguineus* ticks with *E. canis*

*Rhipicephalus sanguineus* ticks, originating from France and maintained for several generations on rabbits under laboratory conditions, were used as a source to generate a batch of ticks infected by *E. canis*. *Rhipicephalus sanguineus* nymphs were fed on a susceptible laboratory-bred Beagle dog, previously inoculated with blood derived from a local case of canine monocytic ehrlichiosis, identified in Bloemfontein, South Africa. After moulting, the adult ticks were used as a basis for the study. A sample taken from the challenge batch of ticks was tested for *E. canis* by PCR and confirmed to be infected at a rate of 17%. The identity of this novel strain of *E. canis* was confirmed by its partial gene sequence (GenBank Accession No. KC935387) of *E. canis* gp36 with a number of other *E. canis* isolates. It was found that *E. canis* (Bloemfontein) is closely related and formed a clade with several Asian isolates [11].

### Tick infestation of dogs

On Days +7, +14, +21 and +28, fifty adult ticks were released into the individual tick-proof kennel of each dog to simulate natural exposure to ticks, instead of applying the ticks directly onto the dogs. The *E. canis*-infected ticks, which were used in the study, were unfed, at least one week old and had a balanced sex ratio of 50% male and 50% female ticks.

### Monitoring of dogs

The study animals were observed on a daily basis between Day −7 and Day +56 for general health purposes. The dogs were observed on an approximately hourly basis for 4 h after initiation of the acaricidal treatment to detect any adverse events. Full clinical examinations were conducted on Days −7, +21, +28, +35 +42, +49 and +56. Rectal body temperatures were recorded daily from Day +17 to Day +56. Additional clinical examinations were conducted on all dogs displaying an abnormally high body temperature (>39.4 °C). Clinical examinations included general appearance, heart rate, respiration rate and body temperature. The examinations focussed on possible clinical manifestations of monocytic ehrlichiosis, which include fever, depression, anorexia, weight loss, haemorrhages and epistaxis. To prevent fatal ehrlichiosis, dogs with abnormally high body temperatures (>39.4 °C) for at least two consecutive days and an abnormally low platelet count were treated with 10 mg/kg doxycycline *per os* for 21 consecutive days.

Blood samples for serology were collected on Days −7, +7, +21, +28, +35, +42, +49 and Day +56 from all dogs. Blood samples for platelet counts and PCR were only collected post tick challenge on Days +21, +28, +35, +42, +49 and Day +56 from all dogs. In dogs with suspected ehrlichiosis (e.g., due to low platelet count), additional samples were taken on the day of diagnosis, before rescue treatment. All samples collected were tested by PCR (Table 4).

### Laboratory tests

Blood samples in EDTA for platelet counts were examined by Pathcare Veterinary Laboratory, Bloemfontein, South Africa. Serum samples were frozen at −20 °C until assayed for *E. canis* antibodies using a commercial IFA test (IGG IFA, Fuller Laboratory, USA). The tests were performed according to the manufacturer’s descriptions at the Department of Veterinary Tropical Diseases (DVTD), Faculty of Veterinary Science, University of Pretoria, South Africa.

A further blood sample collected in EDTA was centrifuged at 3,000 rpm for 15 min and the buffy coat stored in a −80 °C freezer, until PCR assayed. DNA extraction and PCR analysis of all buffy coat samples were performed in the molecular laboratory of ClinVet International Ltd. DNA extractions were performed using Qiagen DNeasy Blood and Tissue kit according to the instructions of the manufacturer. A novel primer set for PCR was designed based on the disulphide oxidoreductase gene of *E. canis*, as previously described [10].

### Tick counts

*In situ* tick thumb counts were carried out on all dogs 48 h after each exposure (Day +9, +16 and +23), but on Day +30 all ticks were counted and removed. The counts were recorded into six categories specified by the “*Guidelines for the Testing and Evaluation of the Efficacy of Antiparasitic Substances for the Treatment and Prevention of Tick and Flea infestation in Dogs and Cats*” adopted on 7 November 2007 by the Committee for Veterinary Medicinal Product of the European Agency for the Evaluation of Medicinal Products (EMEA/CVMP/005/2000-Rev.2). These six categories were: 1 = live, free; 2 = live, attached, unengorged (no filling of the alloscutum); 3 = live, attached, engorged (obvious or conspicuous filling of the alloscutum); 4 = killed, free; 5 = killed, attached, unengorged; 6 = killed, attached and engorged. Ticks counted and removed on Day +30 were also categorised within gender (male/female) in addition to recording them according to categories 1–6. Furthermore, each animal kennel was inspected daily from Day +14 up to Day +30 for any engorged ticks.

### Statistics

In order to determine the effectiveness of the acaricidal treatment, the total number of ticks assigned to counting category 1, 2, 3 and 6 was transformed to the natural logarithm of (count + 1) and then corrected by subtracting one (1) for the calculation of the geometric means. The categories used to calculate effectiveness were according to the recommendations made by the “*Guidelines for the Testing and Evaluation of the Efficacy of Antiparasitic Substances for the Treatment and Prevention of Tick and Flea infestation in Dogs and Cats*” adopted on 7 November 2007 by the Committee for Veterinary Medicinal Product of the European Agency for the Evaluation of Medicinal Products (EMEA/CVMP/005/2000-Rev.2). In the acaricide-treated group, percentage reduction in tick counts compared to the control group was calculated using the formula 100 × (1 − *T*/*C*), wherein *T* and *C* were the geometric means of the acaricide-treated and control group, respectively. Effectiveness was also calculated based on the arithmetic group means. Furthermore, the groups were compared by an ANOVA with a treatment effect after a logarithmic transformation on the (count + 1) tick data.

Dogs which displayed *E. canis* antibodies and were also positive for *E. canis* DNA by PCR analysis were regarded as infected. The proportions of dogs infected in each group were compared by using Fisher’s Exact Test. In addition, the exact 95% confidence interval for the blocking effect in Group 2 was calculated. Version 8 of SAS (Release 8.02 TS Level 02M0) was used for all statistical analyses, whereby the level of significance of the tests was set at 5%.

## Results

### Tick counts

Both arithmetic and geometric mean tick counts recorded for the acaricidal treatment and control groups are provided in Table 1. Statistically significantly (*p* < 0.05) less ticks were recorded on the treated dogs compared to the control dogs on all assessment days. Efficacy values (%) based on mean tick counts for the group treated once are summarised in Table 1. The acaricidal treatment was highly effective (between 96.1% and 98.9%, based on geometric means) against infestations with *R. sanguineus* ticks up to four week post acaricidal treatment.

**Table 1.** Tick counts on dogs 48 h after environmental challenges by treatment group and percentage efficacies.

	Group 1 – Control group						Group 2 – Advantix-treated group						
	Live ticks			Dead ticks			Live ticks			Dead ticks			
Day	Free	Att; Ue	Att; E	Free	Att; Ue	Att; E	Free	Att; Ue	Att; E	Free	Att; Ue	Att; E	Efficacy (%) based on arith. mean (geo. mean#)
+9*	0.0 (0.0)	7.0 (6.7)	0.0 (0.0)	0.0 (0.0)	0.0 (0.0)	0.0 (0.0)	0.0 (0.0)	0.5 (0.3)	0.0 (0.0)	0.0 (0.0)	0.0 (0.0)	0.0 (0.0)	92.9 (96.1)
+16*	0.0 (0.0)	9.5 (8.0)	2.8 (2.1)	0.0 (0.0)	0.0 (0.0)	0.0 (0.0)	0.0 (0.0)	0.2 (0.1)	0.0 (0.0)	0.0 (0.0)	0.0 (0.0)	0.0 (0.0)	98.6 (98.9)
+23*	0.0 (0.0)	10.3 (11.1)	1.8 (1.6)	0.0 (0.0)	0.0 (0.0)	0.0 (0.0)	0.0 (0.0)	0.3 (0.2)	0.0 (0.0)	0.0 (0.0)	0.0 (0.0)	0.0 (0.0)	97.3 (98.3)
+30	0.0 (0.0)	18.5 (17.7)	2.3 (2.1)	0.0 (0.0)	0.5 (0.3)	0.8 (0.7)	0.0 (0.0)	0.5 (0.3)	0.0 (0.0)	0.0 (0.0)	0.0 (0.0)	0.0 (0.0)	97.6 (98.3)

*In situ counts. #The geometric means of Group 2 differed statistically significantly (*p* < 0.05) from those in Group 1 in all cases. Att = Attached; Ue = Unengorged; E = Engorged; Geo = geometric; Arith = Arithmetic.

### *Ehrlichia canis* transmission blocking

There were no adverse effects observed on the dogs with respect to the topical administration of the acaricidal treatment. Three dogs of the control group (CC5 CDA, E46 0EE and CC4 90E) with abnormally high body temperatures (>39.4°C) received doxycycline at 10 mg/kg *per os* for 21 days starting on Day +23 (CC5 CDA), Day +31 (E46 0EE) and Day +38 (CC4 90E). Low platelet counts were observed in the same three dogs with elevated body temperature, but also in a fourth dog of the same group (9B4 937) (Table 2). Thrombocytopenia was evident in all four dogs; as a result of doxycycline treatment, values returned to normal between 200 and 500 × 10^9^/L towards the end of the study on Day +56 (Table 2). In all other animals platelet counts were within the normal range (Table 2).

**Table 2.** Platelet counts in treated and control dogs.

	Platelet counts × 10^9^/L									
Group	Dog no.	Day 21	Day 22	Day 28	Day 30	Day 35	Day 37	Day 42	Day 49	Day 56
Control	CC5 CDA	119	49	–	291	–	–	378	339	372
	CD2 B63	370	–	469	–	343	–	386	394	340
	E46 0EE	337	–	183	5	206	–	370	382	335
	CC4 90E	240	–	284	–	144	97	192	78	256
	CC2 21F	267	–	325	–	259	–	310	190	334
	9B4 937	143	–	36	167	–	–	601	406	508
Treated group	E17 E19	363	–	386	–	295	–	393	395	223
	DF5 A66	338	–	363	–	219	–	344	307	430
	964 441	364	–	411	–	307	–	388	388	451
	CC2 1BD	319	–	391	–	264	–	357	337	353
	CC2 25E	374	–	334	–	263	–	363	321	394
	CC4 55E	262	–	311	–	234	–	321	309	298

The IFA test results are summarised in Table 3. All dogs included in the study tested negative for *E. canis* antibodies prior to the first tick infestation. Four control dogs (Group 1) developed specific antibodies against *E. canis* first detected on Day +21 (2 dogs), Day +28 (one dog) and Day +35 (one dog) and remained positive throughout to the end of the study (Table 3). The same control dogs that developed specific *E. canis* antibodies were confirmed PCR positive. None of the acaricide-treated dogs became seropositive neither PCR positive (Table 4).

**Table 3.** *Ehrlichia canis* antibodies determined by IFA.

Group	Dog no.	Day −7	Day 7^1^	Day 21	Day 28	Day 35	Day 42	Day 49	Day 56
Control	CC5 CDA	–	–2	POS	POS	POS	POS	POS	POS
	CD2 B63	–	–	–	–	–	–	–	–
	E46 0EE	–	–	–	POS	POS	POS	POS	POS
	CC4 90E	–	–	–	–	POS	POS	POS	POS
	CC2 21F	–	–	–	–	–	–	–	–
	9B4 937	–	–	POS	POS	POS	POS	POS	POS
Treated group	E17 E19	–	–	–	–	–	–	–	–
	DF5 A66	–	–	–	–	–	–	–	–
	964 441	–	–	–	–	–	–	–	–
	CC2 1BD	–	–	–	–	–	–	–	–
	CC2 25E	–	–	–	–	–	–	–	–
	CC4 55E	–	–	–	–	–	–	–	–

1 Prior to tick challenge

2 negative; POS = positive.

**Table 4.** Detection of *Ehrlichia canis* DNA by PCR in blood samples from individual dogs.

		PCR									
Group	Dog no.	Day 21	Day 22	Day 23	Day 28	Day 30	Day 35	Day 37	Day 42	Day 49	Day 56
Control	CC5 CDA	POS	POS	POS	nd	nd	nd	nd	nd	nd	nd
	CD2 B63	–2	nd^1^	nd	–	nd	–	nd	–	–	–
	E46 0EE	–	nd	nd	POS	POS	nd	nd	nd	nd	nd
	CC4 90E	–	nd	nd	–	nd	POS	POS	nd	nd	nd
	CC2 21F	–	nd	nd	–	nd	–	nd	–	–	–
	9B4 937	POS	nd	nd	nd	nd	nd	nd	nd	nd	nd
Treated group	E17 E19	–	–	–	–	–	–	–	–	–	–
	DF5 A66	–	–	–	–	–	–	–	–	–	–
	964 441	–	–	–	–	–	–	–	–	–	–
	CC2 1BD	–	–	–	–	–	–	–	–	–	–
	CC2 25E	–	–	–	–	–	–	–	–	–	–
	CC4 55E	–	–	–	–	–	–	–	–	–	–

1 nd = Not done

2 negative; POS = positive.

In total, four out of six dogs became infected with *E. canis* in the control group and none in the acaricide-treated group (*p* = 0.0606).

## Discussion

Advantix^®^ was highly effective (between 96.1% and 98.9%) against infestations of *R. sanguineus* ticks up to four week post acaricidal treatment (Table 1). Previous studies have demonstrated that the efficacy of the 10% w/v imidacloprid/50% w/v permethrin combination at 48 h is above 90%, dependent upon the tick species tested. The acaricidal efficacy of imidacloprid/permethrin spot-on against *R. sanguineus* has been reported to range between 91.5% and 97.6% for up to 37 days [7].

The first study which indicated that the combined action of 10% w/v imidacloprid/50% w/v permethrin could reduce pathogen transmission was published almost a decade ago (3), and reported the prevention of transmission of *A. phagocytophilum* from field-collected *I. scapularis* ticks to dogs treated with Advantix^®^. Another study conducted in southern Italy indicated that the application of 10% w/v imidacloprid/50% w/v permethrin as a topical spot-on reduced *E. canis* infection in dogs [15]. In another study using the same model a fipronil, amitraz and (S)-methoprene combination successfully prevented transmission of *E. canis* by *R. sanguineus* to dogs [10].

Although there was no statistical difference observed between the number of infected dogs in the two study groups, the fact that no infection was observed in Advantix^®^ treated dogs clearly demonstrated that a single administration of Advantix^®^ was able to prevent transmission of *E. canis* by *R. sanguineus* ticks for a duration of 4 weeks. The model that was employed did simulate natural exposure to ticks by releasing them into each individual dog kennel rather than applying them directly onto each dog.

Four out of six control dogs became infected with *E. canis,* as demonstrated by thrombocytopenia, development of specific *E. canis* antibodies and the presence of ehrlichial DNA in blood samples. A possible reason why two out of six control dogs did not develop monocytic ehrlichiosis may have been due to an insufficient number of ehrlichial organisms carried by those ticks that actually fed on these animals, or not all ticks being infected. Although one has to aim for an experimentally infected batch of ticks capable of infecting all control animals, the challenge has to be realistic and comparable with infection rates in field-collected ticks. In this study, 17% of ticks from the batch used to challenge the dogs was infected with *E. canis* as determined by PCR. This infection rate was regarded as comparable and representative for field situations. Although few such studies have been conducted, in general, infection rates with *E. canis* in field ticks are low. For instance, *E. canis* in ticks reported from different endemic areas (either mammalian hosts or questing adults in the environment) varied between 0.09% and 10% [1, 12, 16].

Unfortunately, the presence of *E. canis* by PCR in ticks found on the dogs at the end of the study was not determined. Such data would have provided additional evidence that the dogs had been in contact with infected ticks. Nevertheless, the fact remains that the majority of dogs in the control group (four of six) became infected with *E. canis*, but none of the treated dogs. Since all dogs were challenged with ticks from the same pool of infected ticks, it is very likely that the treated dogs encountered infected ticks as well. None of the six treated dogs became infected with *E. canis,* as confirmed by normal platelet values, lack of specific antibodies and PCR negativity. The results were consistent since the same 4 dogs were thrombocytopenic, seropositive as well as PCR positive (Tables 2–4).

The transmission blocking capacity of Advantix^®^ was complete and provided full protection against monocytic canine ehrlichiosis for 4 weeks post acaricidal treatment.

### Competing interests

The work reported herein was funded by Bayer Animal Health GmbH, Leverkusen, Germany of which D. Stanneck is an employee. ClinVet International Ltd., of which J.J. Fourie is an employee, is a South African Contract Research Organization contracted to carry out the study. F. Jongejan is PhD supervisor of JJF and Professor at the Universities of Pretoria and Utrecht. None of the authors have any personal interest in these studies other than publishing the scientific results that they have been involved in via planning, initiating, monitoring and conducting the investigations and analysing the scientific outcome. Advantix^®^ is a registered trademark of Bayer Animal Health GmbH, Leverkusen, Germany.
